# Ginsenoside Rg3 for Chemotherapy-Induced Myelosuppression: A Meta-Analysis and Systematic Review

**DOI:** 10.3389/fphar.2020.00649

**Published:** 2020-05-12

**Authors:** Linlin Pan, Tingting Zhang, Hongfu Cao, Haiyang Sun, Guirong Liu

**Affiliations:** ^1^Department of Chinese Medicine Literature and Culture, Shandong University of Traditional Chinese Medicine, Jinan, China; ^2^Department of First Clinical Medical College, Shandong University of Traditional Chinese Medicine, Jinan, China; ^3^Institute of Basic Theory of Traditional Chinese Medicine Academy of Chinese Medical Sciences, Beijing, China; ^4^Department of Traditional Chinese Medicine, Shandong University of Traditional Chinese Medicine, Jinan, China

**Keywords:** ginsenoside Rg3, myelosuppression, chemotherapy, systematic review, meta-analysis, randomized-controlled trial (RCT)

## Abstract

Patients with advanced cancer often undergo myelosuppression after receiving chemotherapy. However, severe myelosuppression results in treatment delay, and some can even be life-threatening. At present, cancer patients undergoing chemotherapy urgently need effective intervention strategies to prevent myelosuppression. Fortunately, ginsenoside Rg3 has shown promise as an anti-myelosuppression agent. Therefore, this study was conducted to evaluate the effectiveness of ginsenoside Rg3 in preventing chemotherapy-induced myelosuppression in cancer patients. The PubMed, Cochrane Library, EMBASE, China National Knowledge Infrastructure (CNKI), Weipu (VIP), and Wanfang databases were searched in this study. A total of 18 trials which reported on 2,222 subjects were identified. All trials concerning the use of ginsenoside Rg3 for the prevention of chemotherapy-induced myelosuppression (the decline of leukocyte, hemoglobin, platelet, and neutrophil counts) were randomized-controlled trials. Dichotomous data were expressed as odds ratio (OR) with their respective 95% confidence intervals (CI). The Cochrane evidence-based medicine systematic evaluation was used to evaluate the methodological quality of the included trials. The Review Manager 5.3 and Stata 12.0 software were used to perform the statistical analyses. The trial sequential analysis (TSA) was used to evaluate information size and prevention benefits. The results revealed obvious ginsenoside Rg3-induced improvement in the leukocyte (OR, 0.46; 95% CI, 0.37–0.55), hemoglobin (OR, 0.64; 95% CI, 0.53–0.77), platelet (OR, 0.60; 95% CI, 0.48–0.75) and neutrophil (OR, 0.62; 95% CI, 0.43–0.90) counts at toxic grades I–IV, and leukocyte (OR, 0.39; 95% CI, 0.28–0.54) counts at toxic grades III–IV. The sensitivity analysis revealed that the results were robust. The Egger’s test indicated that there was no publication bias in the results. Overall, this study suggested that ginsenoside Rg3 is beneficial for alleviating the chemotherapy-induced decrease in leukocyte, hemoglobin, platelet, and neutrophil counts. However, the confirmation of the ginsenoside Rg3 can be recommended for myelosuppression patients was limited due to poor methodological quality. Thus, more rigorously designed randomized-controlled trials (RCTs) are required to assess the efficacy of ginsenoside Rg3 for myelosuppression.

## Introduction

Cancer is the most common and lethal disease worldwide, and stress, smoking, and changes in dietary patterns are considered to be among the main causes of this disease (Jemal et al., 2011). Usually, when cancer patients are diagnosed at a middle or late stage, chemoradiotherapy strategies are recommended. Chemotherapy, which is one of the main strategies for advanced cancer, affects the bone marrow microenvironment and hematopoietic growth factors. Intensive chemotherapy may cause severe myelosuppression, which may result in treatment delay, dose reduction, or discontinuation, and thus affect the long-term clinical treatment and lower the survival rate (Bosly et al., 2008; Pettengell et al., 2012). Indeed, some cancer patients may not die from the disease itself, but may die instead from myelosuppression.

Chemotherapy often results in the decline of the leukocyte, erythrocyte (hemoglobin), platelet or neutrophil counts, which often leads to increased susceptibility to the development of infection, bleeding, or anemia in cancer patients (Testart-Paillet et al., 2007; Freifeld et al., 2011). Leukocytes are mainly composed of polynuclear neutrophils and lymphocytes, which are necessary for the defense against bacterial, viral, and parasitic infections. Leukopenia frequently occurs in patients undergoing chemotherapy and is closely monitored due to its potential lethality; neutropenia is the main reason for chemotherapy delay and dose reduction (Silber et al., 1998; Link et al., 2001). Hemoglobin is a major component of red blood cells and an important index reflecting the degree of severity of the anemia. Cancer patients with lower hemoglobin levels displayed a worse response rate and a shorter survival time than those with higher hemoglobin levels (Tampellini et al., 2006). Besides hemostasis, platelets also regulate inflammation. Cancer tumors are described as wounds that do not heal and are characterized by constitutive angiogenesis and inflammation (Desborough et al., 2016). Accordingly, thrombocytopenia is a factor of poor prognosis in patients with hematological malignancy (Gonzalez-Porras et al., 2011; Chen et al., 2012). Therefore, it is necessary to find effective agents that can alleviate chemotherapy-induced myelosuppression.

Pharmacokinetics studies have shown that the common chemotherapeutic agents, such as carboplatin, methotrexate, doxorubicin, etoposide, 5-fluorouracil, paclitaxel, topotecan, and irinotecan, are closely related to myelosuppression (Canal and Chatelut, 1996). Accordingly, reasonable drug selection and medication are critical to reducing chemotherapy-induced myelosuppression. However, there is a lack of effective intervention strategies for preventing myelosuppression in cancer patients undergoing chemotherapy. Fortunately, in recent years, Shenyi capsule has shown promise as an anti-myelosuppression agent.

Shenyi capsule (National Drug Administration Standard Number: Z20030044) is a new anticancer drug developed in China (produced by Jilin Yatai Pharmaceutical Co., Ltd., Changchun, China), with the activity of replenishing qi and blood. Ginsenoside Rg3, the main component of Shenyi capsule, is extracted from ginseng roots. It inhibits tumor growth by obstructing oncogenesis-related pathways, including cell survival, proliferation, invasion, and angiogenesis (Liu et al., 2018). Previous studies have found that ginsenoside Rg3 has inhibitory effects on cancer cell growth and platelet aggregation (Bai et al., 2003), and in combination with chemotherapy prolongs the survival time of patients and reduces adverse chemotherapy-induced reactions (Lee et al., 2012). As a result, in China, ginsenoside Rg3 has been widely used as an adjuvant therapy to chemotherapy in the treatment of various cancers. However, there is no published systematic review and meta-analysis examining the efficacy of ginsenoside Rg3 in preventing chemotherapy-induced myelosuppression. Therefore, for the first time, we conducted a systematic review and meta-analysis based on randomized-controlled trials (RCTs) to evaluate whether ginsenoside Rg3 can prevent the chemotherapy-induced myelosuppression.

## Materials and Methods

This study was conducted according to the Preferred Reporting Items for Systematic Reviews and Meta-Analyses (PRISMA) guidelines, and the data were obtained from published trials.

### Search Strategies

Studies were retrieved from the PubMed, Cochrane Library, EMBASE, China National Knowledge Infrastructure (CNKI), Wanfang and Weipu (VIP) databases. All searches were conducted using MeSH terms and free words. The search period encompassed articles from the established time to October 30, 2019. All the trials were searched regardless of their language and publication type. Two authors (Linlin Pan and Tingting Zhang) independently searched all the related studies in English and Chinese databases using the following search strategies: the following terms were used in Chinese databases: [Shenyi Jiaonang OR Renshen Zaodai Rg3 OR Renshen Zaogan Rg3] AND [Ai OR Exing zhongliu OR Zhongliu] AND [Suiji duizhao shiyan OR Mangfa OR Shuangmang OR Suiji duizhao OR Anweiji]. The following terms were used in English databases: [Ginsenoside Rg3 OR 20s-Ginsenoside Rg3 OR Beta-D- glucopyranoside OR 20(R)] AND [Malignant Neoplasms OR Cancers OR Carcinomas] AND [Randomized controlled trials OR Controlled clinical trial OR Randomized OR Placebo OR Randomly], the detailed search strategy is provided in **Table S1**.

### Inclusion and Exclusion Criteria

#### Inclusion Criteria

The inclusion criteria were the following: (a) participants: we included all patients with any type of cancers who received chemotherapy; (b) interventions: the control group was treated with chemotherapy or chemotherapy plus placebo, the experimental group was treated with chemotherapy combined with ginsenoside Rg3; (c) types of studies: RCTs; (d) outcomes: the decline of the count of leukocytes, hemoglobin, platelets, or neutrophils. In this study, the myelosuppression is graded according to the World Health Organization (WHO) criteria as described in **Table 1**.

**Table 1:** Recommendation for grading of WHO criteria.

Items	0 degree	I degree	II degree	III degree	IV degree
Leukocyte (1000/m^3^)	>4.0	3.0–3.9	2.0–2.9	1.0–1.9	<1.0
Hemoglobin (g/100ml)	>11.0	9.5–10.9	8.0–9.4	6.5–7.9	<6.5
Platelets (1000/m^3^)	>100	75–99	50–74	25–49	<25
Neutrophils (1000/m^3^)	>2.0	1.5–1.9	1.0–1.4	0.5–0.9	<0.5

The exclusion criteria were the following: (a) non-clinical experimental studies (such as reviews, protocols, animal, or cell research studies); (b) patients with other serious blood diseases; (c) patients treated with other antitumor traditional Chinese medicine (TCM) drugs or therapies (such as acupuncture, massage, moxibustion, or Taiji).

### Literature Selection and Data Extraction

The articles were selected from the relevant literature, two independent authors, Linlin Pan and Tingting Zhang, evaluated the title, abstract, and full texts, then selected the relevant trials according to the inclusion criteria, the discrepancies were settled through a consensus discussion. The following information was extracted from the included studies: the name of the first author, year of publication, study types, disease types, stage, sex, age, interventions, detection time, and follow-up time. The outcomes included the levels of leucocyte, hemoglobin, platelet, and neutrophil at toxic grades I–IV and III–IV.

### Risk of Bias in Individual Trials

The methodological quality of each trial was independently assessed by Hongfu Cao and Haiyang Sun using the Cochrane Risk of Bias tool (Higgins et al., 2011). Disagreements were discussed and resolved by Guirong Liu. The following criteria were assessed: random sequence generation, allocation concealment, blinding of participants and personnel, blinding of outcome assessment, incomplete outcome data, selective reporting, and other bias. The risk of bias was classified as “high,” “unclear,” or “low.”

### Data Synthesis and Analysis

The Review Manager Version 5.3 software was used to perform statistical analyses. The odds ratio (OR) with the correspondent 95% confidence intervals (CI) was used to pool the total effectiveness rates of dichotomous data, *P* < 0.05 was considered statistically significant. The χ^2^ and *I^2^* tests were used to evaluate the heterogeneity, the exhibited heterogeneity was *P* < 0.10 or *I^2^* > 50%. The fixed-effect model (FEM) was used for the low heterogeneity data (*P* > 0.10 or *I^2^* < 50%) and the random effects model (REM) was used for the high heterogeneity data (*P* < 0.10 or *I^2^* > 50%). Sensitivity analysis was assessed by re-analyzing the data using various statistical methods. Publication bias was evaluated by visual assessment of the asymmetry of the funnel plots and Egger’s test, where *P* < 0.05 indicated potential bias. The trial sequential analysis (TSA) software (version 0.9.5.10 Beta) was used to calculate the required information size (RIS) for the meta-analysis and evaluate the prevention benefits based on the sample sizes. The risk of type I error was set at 5% with a power of 80%, the variance was calculated based on the data included in the trials, the relative risk reduction was set at 20% (Thorlund et al., ). When the cumulative Z-curves crossed the sequential monitoring boundaries, the evidence for the intervention was sufficient. When Z-curves did not cross the boundaries, the results for the intervention were unreliable (Wetterslev et al., 2017).

## Results

### Search Results

**Figure 1:**
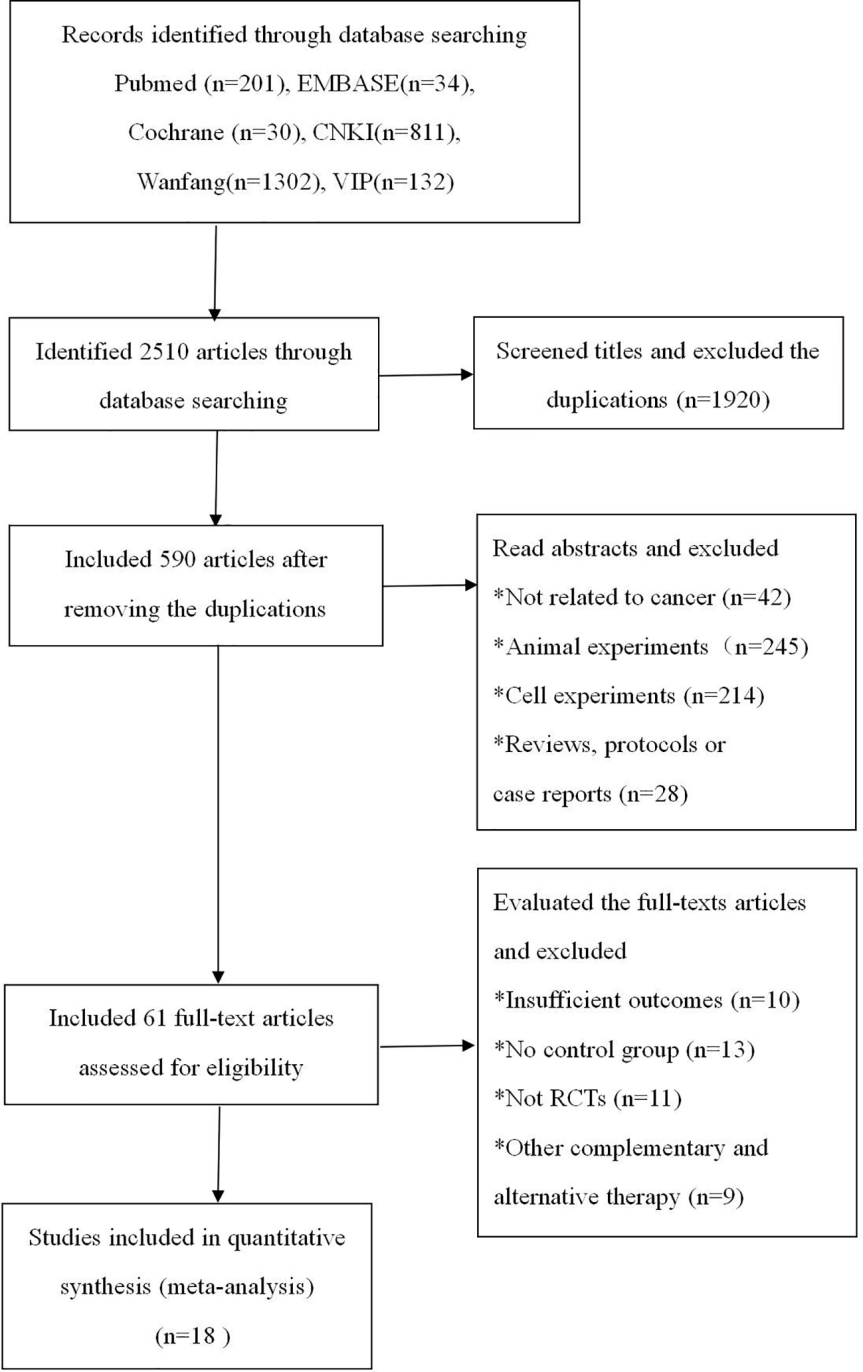
Flow diagram of the literature seaXMrch.

As shown in **Figure 1**, a total of 2,510 studies were identified during the initial database search. First, we used Endnote to exclude 1,920 duplications. Second, we read the abstracts and excluded animal experiment studies (n = 245), cell experiment studies (n = 214), reviews (n = 16), protocols (n = 8), case reports (n = 4), and experimental studies not related to cancer (n = 42). Third, after reading the full-text articles, another 43 studies were excluded due to the following reasons: insufficient outcomes (n = 10), no control group (n = 13), not RCTs (n = 11), and using other complementary and alternative therapy (n = 9). Ultimately, 18 (Shi et al., 2006; Sun et al., 2006; Liu et al., 2007; Liu et al., 2009; Zeng et al., 2009; Zhou et al., 2013; Chen et al., 2014; Dang et al., 2014; Li et al., 2014; Liu et al., 2014; Wang et al., 2014; Wang, 2015; Wei et al., 2015; Zhou et al., 2016; Liu et al., 2017; Zhu, 2017; Tao et al., 2018; Zhang et al., 2018) trials were ultimately included in the analysis.

### Study Characteristics

As shown in **Table 2**, this study included 18 RCTs published from 2,006 to 2,018, comprising 2,222 cancer patients between 18 and 75 years of age. There were 1,154 patients in the experimental groups who underwent ginsenoside Rg3 treatment plus chemotherapy. The dose of ginsenoside Rg3 was 20 mg/time, twice daily. The treatment time per cycle was 3 or 4 weeks, and treatment cycles were two or four cycles. A total of 1,068 patients were included in the control groups and underwent chemotherapy or chemotherapy combined with placebo. Three trials (Sun et al., 2006; Zhu, 2017; Zhang et al., 2018) were multicenter studies, others were single center studies.

**Table 2:** Characteristics of the included studies.

Reference	Year	Study types	Disease types	Stage	Sex (M/F)	Age(years)(E/C)	Interventions		Detection Time	Follow-up time
							E	C		
(Chen et al., 2014)	2014	Randomized, single center	Lung cancer	III–IV	39/29	41–73 (median age 55)	Rg3 +C	TP	12 weeks	3 months
(Dang et al., 2014)	2014	Randomized, single center	Gastric cancer	Advanced stage	Unknown	40–72 (61.06±4.21)/ 40–75 (60.21±4.62)	Rg3 +C	DDP +placebo	8 weeks	8 months
(Li et al., 2014)	2014	Randomized, single center	Lung cancer	Extensive stage	Unknown	Unknown	Rg3 +C	EP	12 weeks	3months
(Liu et al., 2007)	2007	Randomized, double-blind, single center	Lung cancer	Advanced stage	24/11 23/10	65–75 (median age 69)/ 65–75 (median age 70)	Rg3+NP	NP +placebo	6 weeks	6 weeks
(Liu et al., 2017)	2017	Randomized, single center	Gastric cancer	III	29/23 20/17	25–67 (45.1±8.5)/ 27–66 (44.8±9.3)	Rg3 +C	FOLFOX4	12 weeks	6 months
(Liu et al., 2014)	2014	Randomized, double-blind, single center	Lung cancer	III–IV	16/6 15/4	62/ 58 (median age)	Rg3+NP	NP +placebo	6 weeks	2 years
(Liu et al., 2009)	2009	Randomized, double-blind, single center	Lung cancer	III–IV	26/8 19/11	43–75 (median age 62)/ 31–66 (median age 58)	Rg3+NP	NP +placebo	6 weeks	2 years
(Shi et al., 2006)	2006	Randomized, double-blind, single center	Lung cancer	III–IV	16/6 15/4	45–75/37–64	Rg3+NP	NP +placebo	6 weeks	2 years
(Sun et al., 2006)	2006	Randomized, double-blind, multicenter	Lung cancer	III–IV	40/14 39/22	22–75 (median age 62)/ 32–74 (median age 62)	Rg3+NP	NP +placebo	6 weeks	2 years
(Tao et al., 2018)	2018	Randomized, single center	Ovarian cancer	III–IV	150 (female)	37–64 (53.42±14.05)/ 36–63 (53.31±13.98)	Rg3 +C	Gemcitabine	6 weeks	1 year
(Wang, 2015)	2015	Randomized, single center	Lung cancer	III–IV	52/37	38–73 (average age 58.95)	Rg3 +C	NP	6 weeks	6 weeks
(Wang et al., 2014)	2014	Randomized, double-blind, single center	Lung cancer	III	21/11 22/8	65–83 (median age 71)	Rg3 +C	PTX	6 weeks	3 years
(Wei et al., 2015)	2015	Randomized, single center	Gastric cancer	III–IV	68/32 70/30	18–75 (48.5±6.7)/19–74(46.5±5.7)	Rg3 +C	Tegafur +DDP	12 weeks	5 years
(Zeng et al., 2009)	2009	Randomized, single center	Colorectal cancer	IV	25/10 21/11	51–69	Rg3 +C	FOLFOX4	8 weeks	2 months
(Zhang et al., 2018)	2018	Randomized, double-blind, multicenter	Lung cancer	III–IV	128/71 151/64	61.16±10.41/ 60.76±10.39	Rg3 +NP	NP +placebo	6 weeks	3 years
(Zhou et al., 2016)	2016	Randomized, single center	Liver cancer	Advanced stage	128/24 63/13	52.4±11.8/ 52.4±10.4	Rg3 +C	TACE	8 weeks	3 years
(Zhou et al., 2013)	2013	Randomized, double-blind, single center	Gastric cancer	Advanced stage	Unknown	44–73 (median age 61)/ 43–72 (median age 60)	Rg3 +C	PTX +placebo	6 weeks	4 years
(Zhu, 2017)	2017	Randomized, double-blind, multicenter	Cervical cancer	II–IV	302 (female)	<60 (66 patients) ≥60 (86 patients)/ <60 (73 patients) ≥60 (78 patients)	Rg3 +C	TP	8 weeks	1 year

E, experimental group; C, control group; PTX, Paclitaxel; DDP, Cisplatin; FOLFOX4, Oxaliplatin+Calcium folinate+Fluorouracil; TP, Paclitaxel+ Cisplatin; NP, Vinorelbine+ Cisplatin; EP, Etoposide + Cisplatin; TACE, Transcatheter arterial chemoembolization; (1) leukocyte (2) hemoglobin (3) platelet (4) neutrophil.

### Quality Assessment

**Figure 2:**
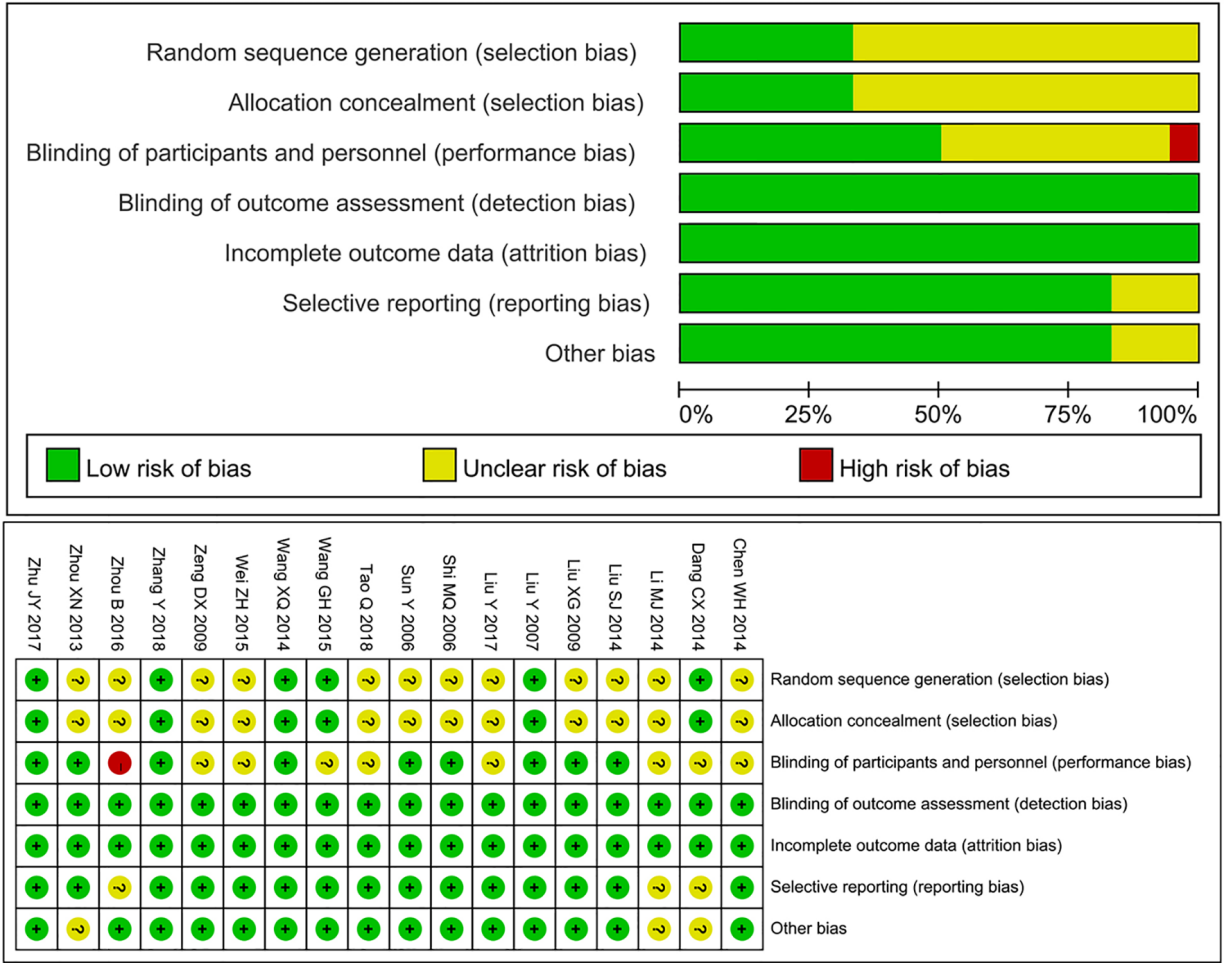
Risk of bias graph.

As shown in **Figure 2**, a total of 18 RCTs were included in this study, the random sequence was generated by using a random number table in four trials (Dang et al., 2014; Wang et al., 2014; Zhu, 2017; Zhang et al., 2018), the single and double randomizations based on the admission number in two trials (Liu et al., 2007; Wang, 2015), others were unclear. A total of nine trials used blind method for participants and personnel (Liu et al., 2007; Liu et al., 2009; Liu et al., 2014; Shi et al., 2006; Sun et al., 2006; Wang et al., 2014; Zhou et al., 2013; Zhu, 2017; Zhang et al., 2018), one trial used open-label trial (Zhou et al., 2016), other trials did not provide detailed information. All trials have a low risk in detection bias, because the grade evaluation of myelosuppression followed the objective (WHO) criteria. In the study by Sun et al. (2006), six patients withdrew from the experimental group, and 13 patients withdrew from the control group (i.e., 16% of patients withdrew from the trial), the remaining trials had complete data without losing follow-up patients, so all trials showed low attrition bias. Regarding reporting bias, three trials (Dang et al., 2014; Liu et al., 2014; Zhou et al., 2016) only reported positive results, so their reporting bias were unclear. As for other bias, except for three trials (Zhou et al., 2013; Dang et al., 2014; Li et al., 2014) were unclear regarding the sex or age of the patients, others had a low risk with the detailed information.

### The Effectiveness of Ginsenoside Rg3 in the Prevention of Chemotherapy-Induced Myelosuppression

#### The Effectiveness of Ginsenoside Rg3 in Improving the Decline of Leukopenia Count

**Figure 3:**
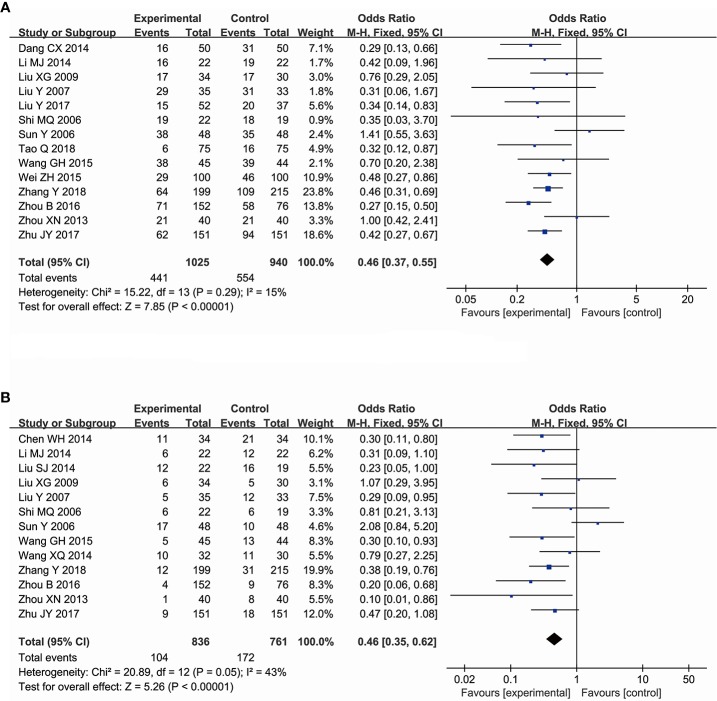
Forest plot for the decline of leukocyte counts. **(A)** The decline of leukocyte counts I–IV. **(B)** The decline of leukocyte counts III–IV.

**Figure 4:**
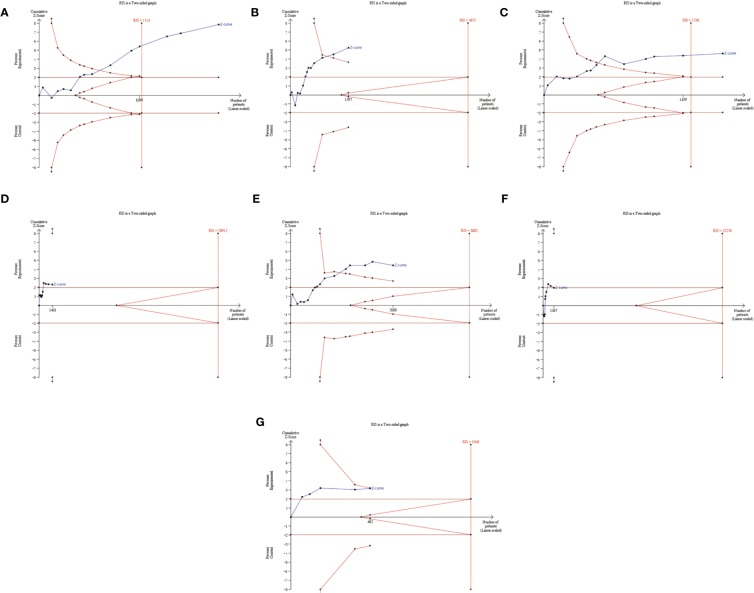
TSA plot for myelosuppression. **(A)** TSA plot for the decline of leukocyte counts (I–IV). (B) TSA plot for the decline of leukocyte counts (III–IV). **(C)** TSA plot for the decline of hemoglobin counts (I–IV). **(D)** TSA plot for the decline of hemoglobin counts (III–IV). **(E)** TSA plot for the decline of platelet counts (I–IV). **(F)** TSA plot for the decline of platelet counts (III–IV). **(G)** TSA plot for the decline of neutrophil counts (I–IV).

A total of 14 trials, comprising 1,965 patients, evaluated the effectiveness of ginsenoside Rg3 in enhancing leukocyte count at toxic grades I–IV induced by chemotherapy, including a total of 1,025 subjects in the experimental group and 940 subjects in the control group. The comparison results showed that ginsenoside Rg3 combined with chemotherapy can reduce leukopenia at toxic grades I–IV in the experimental group (OR, 0.46; 95% CI, 0.37–0.55; *P* < 0.00001 in the Z test). Additionally, the data among the studies showed a low heterogeneity (χ^2^ = 15.22, *P* = 0.19, and *I^2^* = 15%). Therefore, the data were calculated using a FEM (**Figure 3A**). To evaluate the effectiveness of ginsenoside Rg3 in improving leukopenia at toxic grades III–IV, 13 trials comprising 1,597 patients were included. There were 836 subjects in the experimental group and 761 patients in the control group. The pooled data showed that in the experimental group, the effects of ginsenoside Rg3 on leukopenia at toxic grades III–IV was also better than that in the control group (OR, 0.46; 95% CI, 0.35–0.62; *P* < 0.00001 in the Z test), but the results indicated an obvious heterogeneity (χ^2^ = 20.89, *P* = 0.05, and *I^2^* = 43%) (**Figure 3B**). TSA suggested that the accrued information size for the effectiveness of ginsenoside Rg3 in leukopenia at toxic grades I–IV (n = 1,965) was larger than that of RIS (n = 1,124), indicating that the available evidence was sufficient to reach a firm conclusion (**Figure 4A**). However, the accrued information size for leukopenia at toxic grades III–IV (n = 1,597) was 33% of RIS (n = 4,872), indicating that the available evidence was not sufficient to reach a firm conclusion (**Figure 4B**).

#### The Effectiveness of Ginsenoside Rg3 in Improving the Decline of Hemoglobin Count

**Figure 5:**
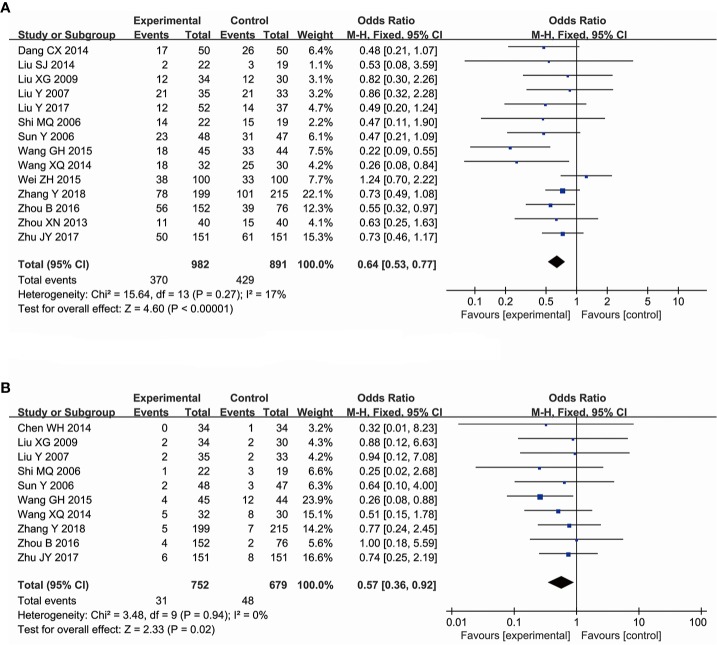
Forest plot for the decline of hemoglobin counts. **(A)** The decline of hemoglobin counts I–IV. **(B)** The decline of hemoglobin counts III-IV.

A total of 14 trials comprising 1,873 patients compared the decline of hemoglobin count among cancer patients, including a total of 982 subjects in the experimental group and 891 subjects in the control group. The comparison revealed that ginsenoside Rg3 combined with chemotherapy was more affective in improving the hemoglobin count at toxic grades I–IV (OR = 0.64, 95% CI: 0.53 to 0.77, and *P* < 0.00001 in the Z test) than chemotherapy alone, and the pooled data showed low heterogeneity (χ^2^ = 15.64, *P* = 0.27, and *I^2^* = 17%), so the data was calculated using a FEM (**Figure 5A**). In the evaluation of the effectiveness of ginsenoside Rg3 in improving the hemoglobin count at toxic grades III–IV, 10 trials, comprising 1,431 patients, were included. There were 752 subjects in the experimental group and 679 subjects in the control group. The pooled data indicated that the enhancing effects of ginsenoside Rg3 on the hemoglobin count at toxic grades III–IV in the experimental group was also stronger than that in the control group (OR = 0.57, 95% CI: 0.36 to 0.92, and *P* = 0.02 in the Z test). The heterogeneity was not observed in the results (χ^2^ = 3.48, *P* = 0.94, and *I^2^* = 0%), thus the data was also calculated using a FEM (**Figure 5B**). TSA suggested that the accrued information size for the effectiveness of ginsenoside Rg3 in improving the hemoglobin count at grades I–IV (n = 1,873) was larger than that of the RIS (n = 1,540). The cumulative Z-curve crossed the trial sequential monitoring boundary, indicating that the available evidence was sufficient to draw a reliable conclusion (**Figure 4C**). The accrued information size for the hemoglobin count at grades III–IV (n = 1431) was 8% of RIS (n = 18912). The cumulative Z-curve did not cross the trial sequential monitoring boundary, indicating that available evidence was not sufficient to draw a reliable conclusion (**Figure 4D**).

#### The Effectiveness of Ginsenoside Rg3 in Improving the Decline of Platelet Count

**Figure 6:**
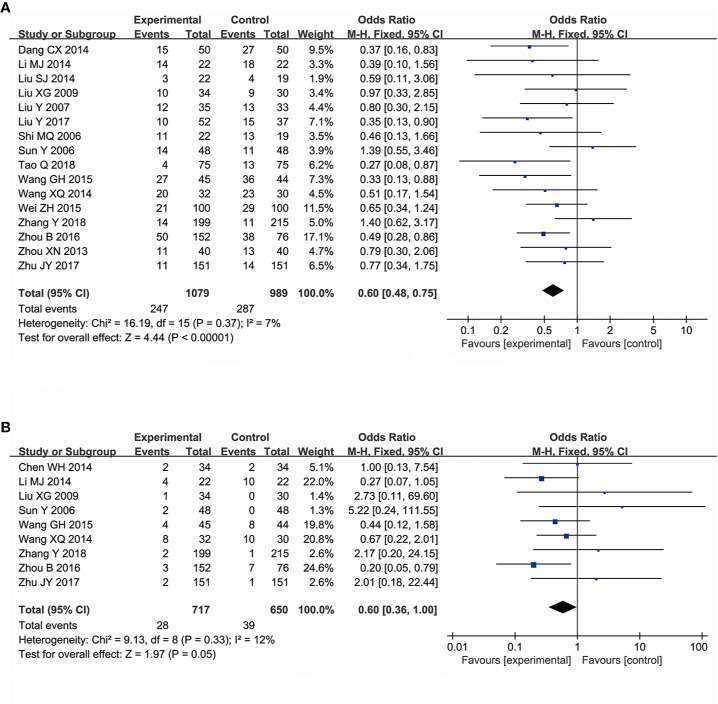
Forest plot for the decline of platelet counts. **(A)** The decline of platelet counts I–IV. **(B)** The decline of platelet counts III–IV.

A total of 16 trials, comprising 2,068 patients, evaluated the decline of the platelet count among cancer patients, including a total of 1,079 subjects in the experimental group and 989 subjects in the control group. The number of patients at toxic grades I–IV with lower decrease of the platelets was observed in the experimental group compared with that in the control group (OR, 0.60; 95% CI, 0.48–0.75; *P* < 0.00001 in the Z test). In addition, a low heterogeneity was observed in the included trials (χ^2^ = 16.19, *P* = 0.37, and *I^2^* = 7%). Therefore, the data was calculated using a FEM (**Figure 6A**). The evaluation of the effectiveness of ginsenoside Rg3 in improving the platelets count at toxic grades III–IV included nine trials comprising a total of 1,367 patients, of which 717 subjects were in the experimental group and 650 subjects were in the control group. However, the effect of ginsenoside Rg3 on improving the platelets count at toxic grades III–IV was not statistically significant (OR, 0.60; 95% CI, 0.36–1.00; *P* = 0.05 in the Z test) (**Figure 6B**). TSA suggested that the accrued information size for the effectiveness of ginsenoside Rg3 in improving the platelet counts at toxic grades I–IV (n = 2,068) was 57% of RIS (n = 3,602) (**Figure 4E**), and for the platelet counts at toxic grades III–IV (n = 1,367) was 8% of RIS (n = 22,276) (**Figure 4F**). Both of their cumulative Z-curves did not cross the trial sequential monitoring boundary, indicating that their current evidence were not sufficient to reach a reliable conclusion.

#### The Effectiveness of Ginsenoside Rg3 in Improving the Decline of Neutrophil Count

**Figure 7:**
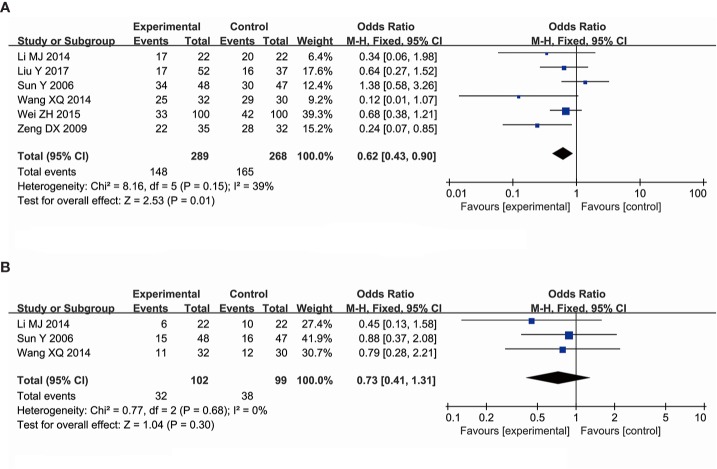
Forest plot for the decline of neutrophil counts. **(A)** The decline of neutrophil counts I–IV. **(B)** The decline of neutrophil counts III–IV.

A total of six trials, comprising a total of 557 patients, evaluated the effectiveness of ginsenoside Rg3 in improving the chemotherapy-induced neutropenia at toxic grades I–IV, including 289 subjects in the experimental group and 268 subjects in the control group. The pooled data showed that ginsenoside Rg3 combined with chemotherapy is more effective in improving neutropenia at toxic grades I–IV (OR, 0.62; 95% CI, 0.43–0.90; *P* = 0.01 in the Z test) compared with chemotherapy alone, while the data indicated an obvious heterogeneity (χ^2^ = 8.16, *P* = 0.15, and *I^2^* = 39%) (**Figure 7A**). In addition, three studies, comprising 201 patients, evaluated the effect of ginsenoside Rg3 on chemotherapy-induced neutropenia, including 102 subjects in the experimental group and 99 subjects in the control group. The results indicated that the suppression of neutrophils at toxic grades III–IV was not statistically significant between the experimental group and control group (OR, 0.73; 95% CI, 0.41–1.13; *P* = 0.30 in the Z test) (**Figure 7B**). TSA suggested that the accrued information size for the effectiveness of ginsenoside Rg3 in improving the neutrophil count at toxic grades I–IV (n = 462, the trial conducted by Sun et al., was ignored) was 44% of RIS (n = 1,046), indicating that the available evidence was not sufficient to reach a firm conclusion (**Figure 4G**).

### Sensitivity Analysis

As shown in **Table 3**, ginsenoside Rg3 can improve the leukocyte and hemoglobin counts at toxic grades I–IV and III–IV, and platelet and neutrophil counts at toxic grades I–IV. However, the change in the effectiveness of ginsenoside Rg3 in improving the platelet and neutrophil counts at toxic grades III–IV was not statistically significant (*P* ≥ 0.05). After excluding the trial conducted by Sun et al. (2006) (**Figure S1**), in which there was not statistically significant difference between the control group and experimental group (*P* > 0.05), the heterogeneity of the leukocyte counts at toxic grades III–IV and neutrophil counts at toxic grades I–IV were significantly reduced, and there was statistically significant difference in the heterogeneity of the platelet counts at toxic grades III–IV. However, no matter which studies were excluded, the data was not statistically significant difference (*P* > 0.05) in the effectiveness of ginsenoside Rg3 in improving the neutrophil counts at toxic grades III–IV.

**Table 3:** Sensitivity analysis.

Analysis	Statistical method		I^2^		Cochran Q		OR (95% CI)			P	
Leukocyte (I–IV)	FEM		15%		P=0.29		0.46[0.37, 0.55]			P < 0.00001	
Hemoglobin (I–IV)	FEM		17%		P=0.27		0.64[0.53, 0.77]			P < 0.00001	
Platelets (I–IV)	FEM		7%		P=0.37		0.60[0.48, 0.75]			P < 0.00001	
Neutrophils (I–IV)	FEM		39%		P=0.15		0.62[0.43, 0.90]			P=0.01	
Leukocyte (III–IV)	FEM		43%		P=0.05		0.46[0.35, 0.62]			P < 0.00001	
Hemoglobin (III–IV)	FEM		0%		P=0.94		0.57[0.36, 0.92]			P=0.02	
Platelets (III–IV)	FEM		12%		P=0.33		0.60[0.36, 1.00]			P=0.05	
Neutrophils (III–IV)	FEM		0%		P=0.68		0.73[0.41, 1.31]			P=0.30	
**Sensitivity analysis via excluding the under- or overestimated trials**											
**Analysis**	**Trials**	**OR (95% CI)**		**I^2^**		**Excluded studies [Reference]**		**Trials**	**OR (95% CI)**		**I^2^**
Neutrophils (I–IV)	6	0.62[0.43, 0.90]		39%		Sun et al., 2006		5	0.51[0.34, 0.78]		10%
Leukocyte (III–IV)	13	0.46[0.35,0.62]		43%		Sun et al., 2006		12	0.38[0.28, 0.52]		0%
Platelets (III–IV)	9	0.60[0.36, 1.00]		12%		Sun et al., 2006		8	0.54[0.31, 0.92]		0%
Neutrophils (III–IV)	3	No statistical significance									

### Publication Bias

**Figure 8:**
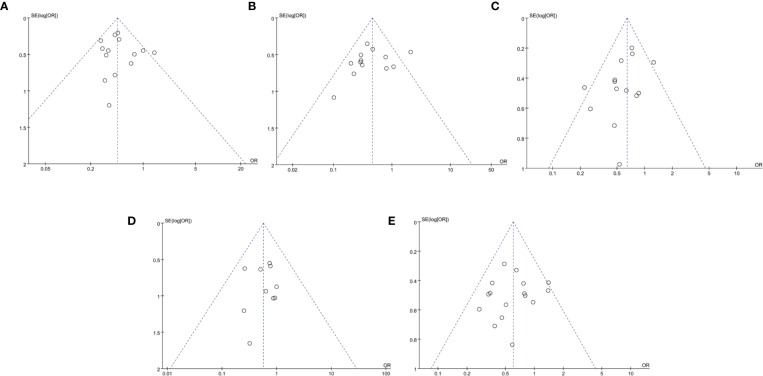
Publication bias plot for myelosuppression. **(A)** Publication bias plot for the decline of leukocyte counts (I–IV). **(B)** Publication bias plot for the decline of leukocyte counts (III–IV). **(C)** Publication bias plot for the decline of hemoglobin counts (I–IV). **(D)** Publication bias plot for the decline of hemoglobin counts (III-IV). **(E)** Publication bias plot for the decline of platelet counts (I–IV).

The funnel plot was nearly symmetrical in the evaluation of the effects of ginsenoside Rg3 on the decline of the counts of leukocytes I to IV (**Figure 8A**) and platelets I to IV (**Figure 8E**), the funnel plot was asymmetrical in the evaluation of the effects of ginsenoside Rg3 on the decline of the counts of leukocytes III to IV (**Figure 8B**), hemoglobin I to IV (**Figure 8C**), and hemoglobin III to IV (**Figure 8D**). Thus, we further used Egger’s test (Stata 12.0) to evaluate their publication bias. As shown in **Table 4**, all of them had a *P* value greater than 0.05, so the Egger’s publication test results suggested that there was no publication bias according to these indicators.

**Table 4:** Egger’s publication test.

Detection Indicators	P value
Leukocyte (I–IV)	0.622
Hemoglobin (I–IV)	0.106
Platelets (I–IV)	0.711
Leukocyte (III–IV)	0.369
Hemoglobin (III–IV)	0.893

## Discussion

### Summary of Reported Evidence

Currently, cisplatin or carboplatin with paclitaxel, gemcitabine, vinorelbine, and cyclophosphamide are commonly used chemotherapeutic agents in the treatment of various cancers. However, the chemotherapy-induced myelosuppression may affect the therapeutic effect of these chemotherapeutic agents. According to TCM theory, ginseng can reinforce vital energy, which is consistent with the enhancement of resistance in modern medicine. The main ingredient of ginseng, ginsenoside Rg3, has antitumor activity, as it can effectively inhibit tumor proliferation by reducing the expression of proliferating cell nuclear antigen, and promote tumor cell apoptosis by increasing the expressions of caspase 3 (Qiu et al., 2019). Additionally, ginsenoside Rg3 can decrease the expression of vascular endothelial growth factor (VEGF), and its antitumor effects may be mediated through suppression of ERK and Akt signaling (Meng et al., 2019). Our prior work indicated that ginsenoside Rg3 combined with chemotherapy can improve the clinical efficacy (objective response rate, disease control rate, survival rate, Karnofsky Performance Scale) and alleviate treatment-induced side effects (gastrointestinal dysfunction, the decline of leucocyte count) for digestive system cancer (Pan et al., 2019). The previous research only suggested that ginsenoside Rg3 can improve the reduction of leucocyte counts in patients with digestive system cancer, but for the chemotherapy-induced decrease in hemoglobin, platelet, and neutrophil counts is unclear. Thus, the objective of this study is to provide powerful evidence that ginsenoside Rg3 can reduce the incidence of myelosuppression induced by chemotherapy in patients with various types of cancers.

Interleukins can be produced by leukocytes and interact with blood cell growth factors to accomplish hematopoiesis and immune regulation. Studies have shown that ginsenoside Rg3 enhances the activity of interleukin 2, and combined with chemotherapy can effectively prevent leukopenia (Wang and Liu, 2015). The hemoglobin count has been reported to have an impact on the chemotherapy outcome in cancers (Bottini et al., 2003). An effective erythropoietic protein (erythropoietin)- mediated hemoglobin response can improve the quality of life of patients undergoing chemotherapy (Desborough et al., 2016). Platelets play a key role in hemostasis, and growing evidence suggests that they are also associated with cancer (Xu et al., 2018). However, chemotherapy treatment in patients with hematological malignancies often impairs the production of platelets (Versteeg et al., 2013). Another earlier study (Zheng et al., 1989) reported that ginseng water extract (containing Rg3) has obvious hemostatic effect and may regulate platelets.

### Summary of Our Evidence

Chemotherapy often results in myelosuppression, which sometimes outweighs its benefits, so how to reduce the chemotherapy-induced myelosuppression while maintaining the effectiveness of the chemotherapeutic agent is a difficult problem that oncologists have been trying to address. In order to evaluate whether ginsenoside Rg3 alleviates chemotherapy-induced myelosuppression in cancer patients undergoing chemotherapy, we conducted the present comprehensive meta-analysis and systematic review of 18 RCTs comprising a total of 2,222 patients. Our search for studies on the inhibitory effect of ginsenoside Rg3 on chemotherapy-induced myelosuppression found that ginsenoside Rg3 was often used in combination with chemotherapy to treat various types of cancers, thus our study also includes a variety of cancers. Compared with chemotherapy alone, ginsenoside Rg3 combined with chemotherapy can significantly improve the decline of leukocyte, hemoglobin, platelet, and neutrophil counts, which confirmed the effectiveness of ginsenoside Rg3 in alleviating chemotherapy-induced myelosuppression in patients with various types of cancers.

We have rigorously screened and controlled the included trials. In the quality assessment, the 18 trials included were RCTs, but only one-third of the trials showed low risk in selection bias, and half of them were of low risk in performance bias. Fortunately, the detection bias risk and attrition bias risk were low in all trials, and most trials had low risk in reporting bias and other bias. In the publication bias, the funnel plots combined with Egger’s test showed that the results had no publication bias (*P* > 0.05). In the sensitivity analysis, after excluding the trial of Sun et al., 2006, the results showed that the number of myelosuppression (I–IV and III–IV) in the experimental group was significantly lower than that in the control group, so the data consistency of this study is generally good. TSA suggested that the available evidence of leukocyte I–IV and hemoglobin I–IV were sufficient to reach a reliable conclusion, but the accrued information size for the remaining indicators failed to meet the RIS. In addition, the pooled data from the RCTs suggested that the optimal dose of ginsenoside Rg3 combined with chemotherapy were 20 mg/time, twice daily. The treatment time per cycle was 3 or 4 weeks, and treatment cycles were two or four cycles.

It is generally recognized that the average survival time of granulocytes is about 6 to 8 h, so the first manifestation of myelosuppression is leukopenia. Previous study reported the relationship between leukocyte counts and overall survival, showing that patients with leukocyte counts below 4 × 10 (9)/L had worse overall survival (Pei et al., 2014). In this study, the pooled data also indicated that ginsenoside Rg3 can alleviate leukopenia at toxic grades I–IV and III–IV, which further verified that ginsenoside Rg3 can inhibit the leukopenia induced by chemotherapy.

In addition, myelosuppression is graded according to the anticancer drugs in the acute and subacute toxicity of the classification criteria (WHO criteria). Generally, patients with chemotherapy-induced degree III–IV myelosuppression must be treated. In the trials included in this study, the pooled data indicated the great effectiveness of ginsenoside Rg3 in alleviating the leukopenia at toxic grades III–IV. However, due to the small number of RCTs, we cannot accurately determine whether ginsenoside Rg3 can improve the decline of hemoglobin, platelet, and neutrophil counts at toxic grades III–IV induced by chemotherapy. Thus, more high quality RCTs are required to make an accurate assessment in this regard.

### The Limitation and Expectation

This study still had the following limitations. First, ginsenoside Rg3 is an important component of ginseng, which is widely used in China for the treatment of cancers. In this study, we reviewed studies performed and published in China and English-speaking countries, 17 studies were published in Chinese, and one study was published in English. Thus, the included trials may lead to geographical bias to some extent, so we anticipate that there will be more RCTs in other countries to further confirm the effectiveness of ginsenoside Rg3 in alleviating chemotherapy-induced myelosuppression in cancer patients undergoing chemotherapy. Second, some of the included studies were unclear in the allocation concealment and blinding method used. Since the included research indicators were mostly followed by the WHO criteria, they were unlikely to have a serious impact on the assessment, although we still hope that researchers can describe the experimental scheme in more detail.

## Conclusion

This meta-analysis and systematic review provided useful information for clinicians, indicating that ginsenoside Rg3 can alleviate the chemotherapy-induced reduction of the counts of leukocyte, hemoglobin, platelet, and neutrophil. However, the role of ginsenoside Rg3 in improving severe myelosuppression needs to be further explored, and the confirmation of ginsenoside Rg3 can be recommended for myelosuppression patients was limited due to the poor methodological quality. In future studies, more rigorously designed RCTs are required to verify the above findings.

## Data Availability Statement

All data sets generated for this study are included in the article/**Supplementary Material**.

## Author Contributions

Conception and design, development of methodology by LP and TZ. Literature search, article selection, and data extraction by LP and TZ. The assessment of methodological bias risk and statistical analysis by HC and HS. Preparation of the manuscript draft by LP. Study supervision, review, and revision of the manuscript by GL. All authors read and approved the final version of the manuscript.

## Funding

This work was supported by The State Administration of Traditional Chinese Medicine, Master of Traditional Chinese Medicine Zhang Zhiyuan Studio Construction Project (National Chinese Medicine Office Letter [2018], National Traditional Chinese Medicine Administration Key Discipline Construction Project of Traditional Chinese Medicine (National Chinese Medicine Office Letter [2012] No. 32).

## Conflict of Interest

The authors declare that the research was conducted in the absence of any commercial or financial relationships that could be construed as a potential conflict of interest.
