# Supplementary figures and images for: Ginsenoside Rg3 for Chemotherapy-Induced Myelosuppression: A Meta-Analysis and Systematic Review
Source: Front Pharmacol. 2020 May 12;11:649. doi: 10.3389/fphar.2020.00649 (PMC7235324; doi:10.3389/fphar.2020.00649)

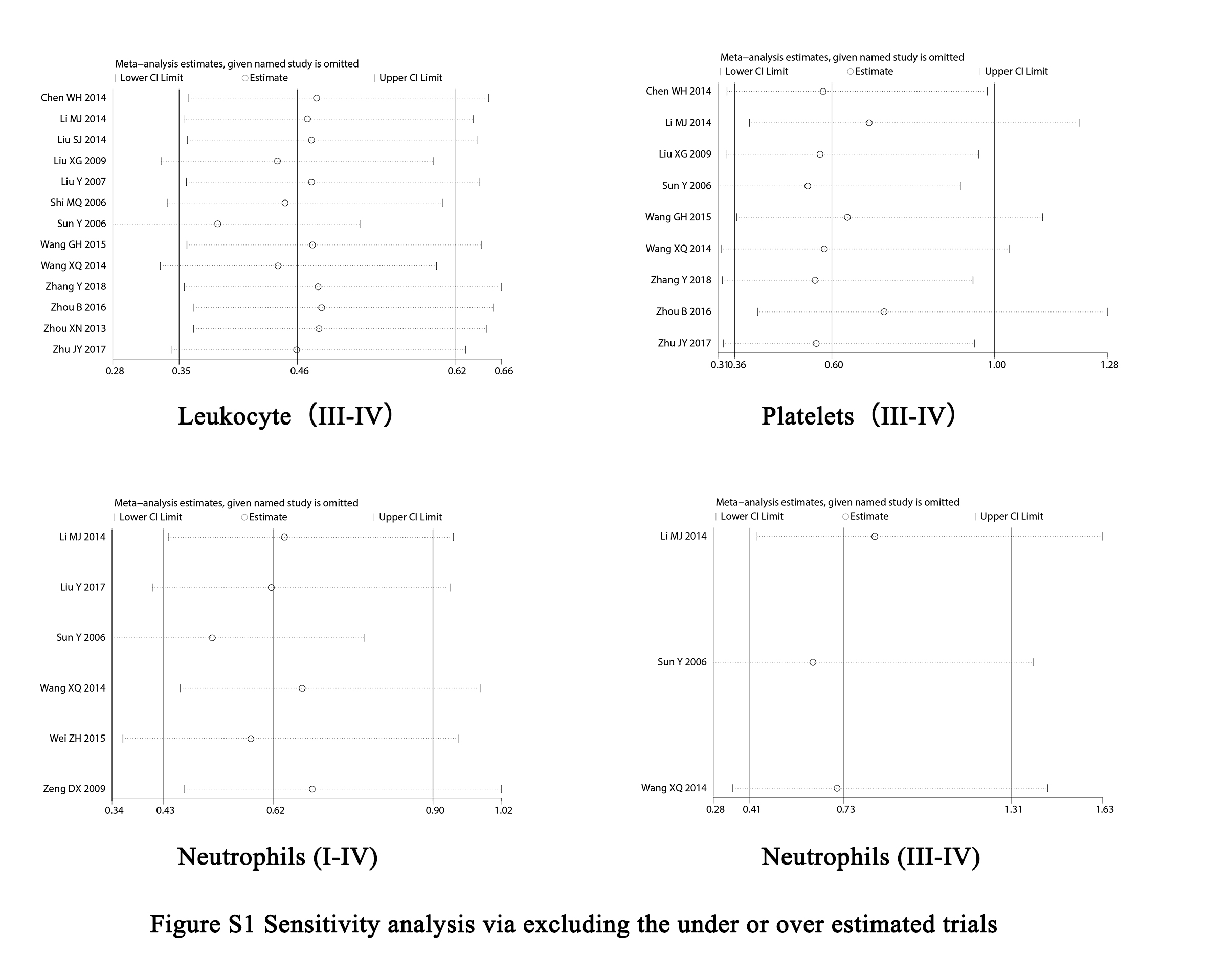

Supplement: Supplementary file 1 [file Image_1.jpeg]
