# Supplementary material for: Ginsenoside Rg3 for Chemotherapy-Induced Myelosuppression: A Meta-Analysis and Systematic Review
Source: Front Pharmacol. 2020 May 12;11:649. doi: 10.3389/fphar.2020.00649 (PMC7235324; doi:10.3389/fphar.2020.00649)
Supplement: Supplementary file 2 [file DataSheet_1.doc]

**Pubmed**

| Recent queries in pubmed | | |
| --- | --- | --- |
| Search | Query | Items found |
| #10  （#3 AND #6 AND 9） | Search((((((((((((3-O-beta-D-glucopyranosyl-(1->2)-beta-D-glucopyranosyldammar-24-ene-3beta,12beta,20s-triol[Title/Abstract]) OR 20s-ginsenoside rg3[Title/Abstract]) OR 20(S)-ginsenoside Rg(3)[Title/Abstract]) OR s-ginsenoside rg3[Title/Abstract]) OR ginsenoside rg3[Title/Abstract]) OR beta-D-glucopyranoside, (3beta,12beta)-12,20-dihydroxydammar-24-en-3-yl 2-O-beta-D-glucopyranosyl-[Title/Abstract]) OR ginsenoside 20-rg3[Title/Abstract]) OR 20(R)-ginsenoside Rg(3)[Title/Abstract])) OR "ginsenoside Rg3" [Supplementary Concept])) AND (("Neoplasms"[Mesh]) OR (((((((((cancer[Title/Abstract]) OR Neoplasia[Title/Abstract]) OR Neoplasm[Title/Abstract]) OR Tumor[Title/Abstract]) OR Malignancy[Title/Abstract]) OR Malignant Neoplasms[Title/Abstract]) OR Neoplasm, Malignant[Title/Abstract]) OR Benign Neoplasms[Title/Abstract]) OR Neoplasm, Benign[Title/Abstract]))) AND (("Random Allocation"[Mesh]) OR (((((Randomized controlled trials) OR Controlled clinical trial) OR Randomized allocation) OR placebo) OR randomly)) | 2201 |
| #9  （#7 OR #8） | Search ("Random Allocation"[Mesh]) OR (((((Randomized controlled trials) OR Controlled clinical trial) OR Randomized allocation) OR placebo) OR randomly) | 1092041 |
| #8 | Search ((((Randomized controlled trials) OR Controlled clinical trial) OR Randomized allocation) OR placebo) OR randomly | 1092041 |
| #7 | Search "Random Allocation"[Mesh] Sort by: [pubsolr12] | 101924 |
| #6  （#4 OR #5） | Search ("Neoplasms"[Mesh]) OR (((((((((cancer[Title/Abstract]) OR Neoplasia[Title/Abstract]) OR Neoplasm[Title/Abstract]) OR Tumor[Title/Abstract]) OR Malignancy[Title/Abstract]) OR Malignant Neoplasms[Title/Abstract]) OR Neoplasm, Malignant[Title/Abstract]) OR Benign Neoplasms[Title/Abstract]) OR Neoplasm, Benign[Title/Abstract]) | 3995199 |
| #5 | Search ((((((((cancer[Title/Abstract]) OR Neoplasia[Title/Abstract]) OR Neoplasm[Title/Abstract]) OR Tumor[Title/Abstract]) OR Malignancy[Title/Abstract]) OR Malignant Neoplasms[Title/Abstract]) OR Neoplasm, Malignant[Title/Abstract]) OR Benign Neoplasms[Title/Abstract]) OR Neoplasm, Benign[Title/Abstract] | 2466135 |
| #4 | Search "Neoplasms"[Mesh] Sort by: [pubsolr12] | 3276408 |
| #3  （#1 OR #2） | Search (((((((((3-O-beta-D-glucopyranosyl-(1->2)-beta-D-glucopyranosyldammar-24-ene-3beta,12beta,20s-triol[Title/Abstract]) OR 20s-ginsenoside rg3[Title/Abstract]) OR 20(S)-ginsenoside Rg(3)[Title/Abstract]) OR s-ginsenoside rg3[Title/Abstract]) OR ginsenoside rg3[Title/Abstract]) OR beta-D-glucopyranoside, (3beta,12beta)-12,20-dihydroxydammar-24-en-3-yl 2-O-beta-D-glucopyranosyl-[Title/Abstract]) OR ginsenoside 20-rg3[Title/Abstract]) OR 20(R)-ginsenoside Rg(3)[Title/Abstract])) OR "ginsenoside Rg3" [Supplementary Concept] | 331 |
| #2 | Search (((((((3-O-beta-D-glucopyranosyl-(1->2)-beta-D-glucopyranosyldammar-24-ene-3beta,12beta,20s-triol[Title/Abstract]) OR 20s-ginsenoside rg3[Title/Abstract]) OR 20(S)-ginsenoside Rg(3)[Title/Abstract]) OR s-ginsenoside rg3[Title/Abstract]) OR ginsenoside rg3[Title/Abstract]) OR beta-D-glucopyranoside, (3beta,12beta)-12,20-dihydroxydammar-24-en-3-yl 2-O-beta-D-glucopyranosyl-[Title/Abstract]) OR ginsenoside 20-rg3[Title/Abstract]) OR 20(R)-ginsenoside Rg(3)[Title/Abstract] | 331 |
| #1 | Search "ginsenoside Rg3" [Supplementary Concept] Sort by: [pubsolr12] | 322 |

**Embase**

| No. | Query | Results |
| --- | --- | --- |
| #23 | ('ginsenoside rg 3'/exp OR '20s-ginsenoside rg3':ab,ti OR '20(s)-ginsenoside rg(3)':ab,ti OR 's-ginsenoside rg3':ab,ti OR 'ginsenoside rg3, (+)-':ab,ti OR 'ginsenoside rg3, (s)-':ab,ti OR 'beta-d-glucopyranoside':ab,ti OR 'ginsenoside 20-rg3':ab,ti OR '20(r)-ginsenoside rg(3)':ab,ti) AND ('malignant neoplasm'/exp OR 'cancer':ab,ti OR 'neoplasia':ab,ti OR 'neoplasm':ab,ti OR 'tumor':ab,ti OR 'malignancy':ab,ti OR 'neoplasm, malignant':ab,ti) AND ('random':ab,ti OR 'placebo':ab,ti OR 'double-blind':ab,ti) | 34 |
| #22 | 'random':ab,ti OR 'placebo':ab,ti OR 'double-blind':ab,ti | 666898 |
| #21 | 'double-blind':ab,ti | 191315 |
| #20 | 'placebo':ab,ti | 299884 |
| #19 | 'random':ab,ti | 314296 |
| #18 | 'malignant neoplasm'/exp OR 'cancer':ab,ti OR 'neoplasia':ab,ti OR 'neoplasm':ab,ti OR 'tumor':ab,ti OR 'malignancy':ab,ti OR 'neoplasm, malignant':ab,ti | 4751081 |
| #17 | 'neoplasm, malignant':ab,ti | 52 |
| #16 | 'malignancy':ab,ti | 211398 |
| #15 | 'tumor':ab,ti | 1533146 |
| #14 | 'neoplasm':ab,ti | 77115 |
| #13 | 'neoplasia':ab,ti | 72289 |
| #12 | 'cancer':ab,ti | 2327328 |
| #11 | 'malignant neoplasm'/exp | 3622674 |
| #10 | 'ginsenoside rg 3'/exp OR '20s-ginsenoside rg3':ab,ti OR '20(s)-ginsenoside rg(3)':ab,ti OR 's-ginsenoside rg3':ab,ti OR 'ginsenoside rg3, (+)-':ab,ti OR 'ginsenoside rg3, (s)-':ab,ti OR 'beta-d-glucopyranoside':ab,ti OR 'ginsenoside 20-rg3':ab,ti OR '20(r)-ginsenoside rg(3)':ab,ti | 1735 |
| #9 | '20(r)-ginsenoside rg(3)':ab,ti | 20 |
| #8 | 'ginsenoside 20-rg3':ab,ti | 1 |
| #7 | 'beta-d-glucopyranoside':ab,ti | 628 |
| #6 | 'ginsenoside rg3, (s)-':ab,ti | 6 |
| #5 | 'ginsenoside rg3, (+)-':ab,ti | 489 |
| #4 | 's-ginsenoside rg3':ab,ti | 63 |
| #3 | '20(s)-ginsenoside rg(3)':ab,ti | 38 |
| #2 | '20s-ginsenoside rg3':ab,ti | 2 |
| #1 | 'ginsenoside rg 3'/exp | 1042 |

**Cochrane**

| ID | Search | Hits |
| --- | --- | --- |
| #1 | MeSH descriptor: [Ginsenosides] explode all trees | 52 |
| #2 | MeSH descriptor: [Neoplasms] explode all trees | 77072 |
| #3 | (Neoplasia):ti,ab,kw (Word variations have been searched) | 2908 |
| #4 | (cancer):ti,ab,kw (Word variations have been searched) | 159283 |
| #5 | (Malignancy):ti,ab,kw (Word variations have been searched) | 26469 |
| #6 | (tumor):ti,ab,kw (Word variations have been searched) | 73709 |
| #7 | (ginsenoside rg3):ti,ab,kw (Word variations have been searched) | 30 |
| #8 | (20s-ginsenoside rg3):ti,ab,kw (Word variations have been searched) | 0 |
| #9 | (s-ginsenoside rg3):ti,ab,kw (Word variations have been searched) | 1 |
| #10 | #1 OR #7 OR #8 OR #9 | 75 |
| #11 | #2 OR #3 OR #4 OR #5 OR #6 | 302443 |
| #12 | #10 AND #11 | 30 |

**CNKI (in the advanced search)**

| ID | Search | Hits |  |
| --- | --- | --- | --- |
| #1 | MeSH: Shenyi Jiaonang OR Renshen zaodai Rg3 OR Renshen zaogan Rg3 | | 3462 |
| #2 | #1 AND (Ai OR Exing zhongliu OR Zhongliu) | | 1824 |
| #3 | #2 AND (Suiji duizhao shiyan OR Mangfa OR Shuangmang OR Suiji duizhao OR Anweiji) | | 811 |

**Wangfang (in the advanced search)**

| ID | Search | Hits |  |
| --- | --- | --- | --- |
| #1 | MeSH: Shenyi Jiaonang OR Renshen zaodai Rg3 OR Renshen zaogan Rg3 | | 4820 |
| #2 | #1 AND (Ai OR Exing zhongliu OR Zhongliu) | | 2074 |
| #3 | #2 AND (Suiji duizhao shiyan OR Mangfa OR Shuangmang OR Suiji duizhao OR Anweiji) | | 1302 |

**VIP (in the advanced search)**

| ID | Search | Hits |  |
| --- | --- | --- | --- |
| #1 | MeSH: Shenyi Jiaonang OR Renshen zaodai Rg3 OR Renshen zaogan Rg3 | | 723 |
| #2 | #1 AND (Ai OR Exing zhongliu OR Zhongliu) | | 367 |
| #3 | #2 AND (Suiji duizhao shiyan OR Mangfa OR Shuangmang OR Suiji duizhao OR Anweiji) | | 132 |
